# Progress in Medicine

**Published:** 1896-02-22

**Authors:** 


### Progress in Medicine.

### ON DIPHTHERITIC NEURITIS.

**Age of Patients Affected.**—The 125 cases reported by Goodall[1] show 96 under 10 years, and 25 aged from 10 to 20, and 71 were between the ages of 3 and 8 years. These figures are significant as showing that though infants escape (apparently), we must remember that the symptoms of neuritis are difficult to demonstrate in infants unless the paralyses are very marked, and that almost all the severe cases in infants die. Goodall's statistics, therefore, confirm those of Ross and Bury[2] that paralysis is more common in children than in adults, and that the liability decreases with age. These observations are quite at variance with those of Landouzy, quoted by Gowers,[3] that "adults furnish a larger proportion of the cases of paralysis than of diphtheria." No doubt Landouzy's observations were correct, but the error he made was not to subtract the cases that died before paralysis was established. If this is done, the percentage of paralysis under 10 years old is 22 per cent.; between

10 and 20 years, 15·1 per cent.; above 20 years, only 3·7 per cent.

**Number of Cases.**—The frequency with which diphtheria is followed by paralysis has been most variously stated; varying, in fact, in different epidemics, from 8 to 66 per cent. (Gowers). Goodall's cases, 1,071 in all, gave 125 cases of paralysis, a proportion of 11·6 per cent., which does not differ from Eustace Smith's[4] estimate of one in every 10 or 12 cases of diphtheria (taken from Greenfield). But Goodall points out that we ought rather to estimate the chances of paralysis by deducting the cases which die early before paralysis is declared, which gives an incidence of paralysis of 17·6 per cent.

Are mild or severe cases of diphtheria more commonly followed by paralysis?

Henoch[5] said, "Diphtheritic paralysis occurs most commonly on the milder attacks of the disease." Eustace Smith and Gowers agree that mild cases are as likely to be affected as severe ones. Ross declines to commit himself on the point, but is content with the remark that slight sore throat may be followed by well-marked paralysis. Goodall does not deny the latter statement, but his cases prove conclusively that the greater the amount and duration of the membrane the greater is the liability to paralysis, a conclusion that Washbourne[6] emphatically endorses. The observations of Cadet de Gassicourt[7] do not differ from Goodall.

**Albuminuria.**—"The amount and duration of the albuminuria give a fair idea of the degree of poisoning by the toxic products of the bacillus" (Goodall); hence we find in his 1,071 cases 30 per cent. had albuminuria, but of the 125 cases of paralysis 110; 88 per cent. passed albumen, and 70·4 of this 110 passed it for more than a week. Goodall classified 22 cases of paralysis as severe of which 13 died; all had albuminuria, 10 of them excessive. Of these 13 fatal cases the exudation persisted from 6 to 33 days.

**Death Rate.**—The proportion of deaths from diphtheritic neuritis is very doubtful. Bristowe, Eustace Smith, and Hilton Fagge are all agreed that deaths from this condition are extremely rare, but the assumption is a dangerous one to adopt. From 1885 to 1891 twenty-nine cases were treated at Paddington Green Hospital, of whom eight died (27·4 per cent.). Deduction from such a small number is not safe, but undoubtedly many cases of death after diphtheria which are attributed, from their clinical aspect, to capillary bronchitis and "cardiac failure" are in reality due to bulbar or pneumo-gastric poisoning.

In the last few years attention has been drawn to the important fact that suppression of urine may take place. This anuria is always heralded by albuminuria, and the patient may appear to be improving rapidly, even though almost total suppression has existed for many hours. In such cases death may be very rapid. It is of as much moment to determine the amount of urine passed daily as the presence of albumen; in fact, a sudden diminution of albumen may accompany a steady diminution of urine. Vomiting in such cases usually heralds death. There is no coma, and convulsions are rare. The pulse usually falls from its previous rapid rate; but this fall may be followed by a sudden rapid rise and great irregularity.

The sensory disturbances of diphtherial neuritis were not clearly recognised for a long time, probably because such a large proportion of cases were young children, in whom a slight degree of numbness or other paræsthesia cannot be well determined. Pain and sense of soreness in the distribution of the affected nerve trunks is very common (Bristowe, p. 348; Ross, p. 254). Moreover, just as the paralytic symptoms are remarkable for their tendency to symmetry of distribution, so also are patches of anæsthesia limited to the middle line of the body. (Bristowe). Numbness, tingling, and anæsthesia are as frequently present in the palate fauces and tongue as paresis is in these localities. Muscular sense is often diminished or lost (Ross).

In three cases Ross detected "amaurosis" apart from ciliary and oculomotor paresis. He thinks it probable that this was due to optic neuritis. Bouchut described a serous infiltration of the retina, and in exceptional cases a true optic neuritis, but this has never been confirmed by other observers.

**Knee Jerks.**—It has been known since 1880 that the patellar reflexes frequently disappear for a time, and this absence of knee jerk may be the only symptom of neuritis. This absence of knee jerk is often preceded by an exaggeration of jerk lasting several days (Money, Herringham, Barlow); the increased jerks may be maintained throughout the whole period of paralysis, and only disappear with the clearing up of the paralysis (Bristowe, "Diseases of the Nervous System," p. 350).

**Bulbar Crises.**—Sudden heart failure may be due either to fatty degeneration of the cardiac muscle itself, which is found frequently in animals injected with diphtheritic toxin,[10] to peripheral degeneration of cardiac nerves, or to bulbar neuritis. These bulbar cases have been minutely studied at the Paddington Green Children's Hospital by Guthrie,[9] and at the North-Eastern Children's by Pasteur.[11] The patients are usually first seen in the second or third week of diphtheritic paralysis; they then present slight paresis of the legs ("they tumble about"), nasal voice, paresis of palate, and sometimes squint. The voice is, or may suddenly become, weak and hoarse, and their cough is loose, ineffectual, and noiseless.[12] This combination of symptoms in children, probably due to bilateral paresis of the vocal cords, is unmistakable when once seen. The respiration often gives warning of approaching danger; it is not rapid, but the inspiration is sudden, deep, and forcible, and expiration short and weak (Landouzy, Guthrie); mucus accumulates readily and rapidly in the fauces and air passages. The crises which occur, and which are so fatal, are marked by sudden and complete paralysis of deglutition and complete aphonia, with most alarming dyspnœa. The pulse rate rises to 140 or 150, and the temperature may rise up to 102 or 103 degrees. Another symptom of these crises is uncontrolled vomiting: this was ascribed by Gee and Sympson[13] to uræmia, but only two of Guthrie's cases presented any large amount of albuminuria, and it is more reasonable to suppose that vagus neuritis is the cause. These crises tend to recur, and the periods of intermission and degree of relief vary; they may last from a few minutes to several hours. Even when 48 hours have elapsed since a crisis it is impossible to say that further attacks may not occur and kill the patient. The proportion of recovery in such attacks is very small.

Pasteur devoted much attention to the state of the lungs and respiratory muscles in such crises. Paralysis of the diaphragm occurred in 28 of 32 cases, and in four of these the intercostal muscles were also affected. These conditions need not be bulbar, but were probably due to neuritis of the phrenic and intercostal nerves. The bulbar form of phrenic paralysis is very sudden in onset, and the peripheral form more insidious. Obviously the former is more dangerous.

Clinically such cases simulate acute broncho-pneumonia, and such a case was described twenty years ago[14] as "a case of diphtheritic paralysis simulating extensive lung disease." Any medical man seeing such a case at the height of a crisis could hardly avoid diagnosing it as capillary bronchitis and cardiac syncope, especially if there were no clear history of diphtheria; but, as Guthrie points out, children with bronchitis do not seem well within a few hours of death, nor do they die of syncope from these causes until many days after they have been obviously and seriously ill.

**Repeated Vomiting** has been long recognised as a sign of bad augury. Gee "collected nine cases, fatal all but one. There need be no suspicion in these cases of membrane in the stomach, which is of extreme rarity, though not unknown." Vomiting is so often associated with paresis of the laryngeal muscles and alteration in the action of the heart that at present we are justified in regarding it as due to affection of the vagus centres or trunk.

An important question, to which we have sought an answer, is whether diphtheritic paralysis which has recovered ever recurs. The authorities we have searched seem agreed that peripheral neuritis does not recur, but, as we have seen, the crises due to poisoning of the medulla may be recurrent for many days, and must, therefore, give the gravest apprehension.

[1] Goodall—"Brain," 1895, p. 284. [2] Ross and Bury—"Peripheral Neuritis," p. 240. [3] Gowers—"Dis. of Nervous Syst.," 2nd Ed., II., p. 903. [4] E. Smith—"Dis. in Childhood," p. 99. [5] Henoch—"Lectures on Children's Diseases," Syd. Soc. Trans., Vol. II. p. 300. [6] Washbourne—Brit. Med. Journ., 1895, II., 411. [7] Cadet de Gassicourt—"Traité Clinique des Maladies de l'Enfance," Vol. III., p. 342. [8] "Diseases of Nervous System." [9] Guthrie—Lancet, 1891, I. [10] Sydney Martin—Brit. Med. Journ., 1895, II., 462. [11] W. Pasteur—Clinical Soc. Trans., XXVIII. [12] Gee—St. Barthol. Hosp. Rep., XXV. [13] Sympson—Practitioner, Jan., 1891. [14] Pearson Irvine—Trans. Clinical Soc., 1876.

